# Addressing COVID-19 Rumors and Behaviors Using Theory in Guyana: A Program Case Study

**DOI:** 10.9745/GHSP-D-22-00071

**Published:** 2022-08-30

**Authors:** Bolanle Olapeju, Camille Adams, Joann Simpson, Lyndsey Mitchum, Sean Wilson, Mona Jarrah, Gabrielle Hunter, TrishAnn Davis, Alicia Martin, Shabana Shaw, Natalie Tibbels, Jennifer Orkis, J. Douglas Storey

**Affiliations:** a Johns Hopkins Center for Communication Programs, Baltimore, MD, USA.; b Department of Health, Behavior and Society, Johns Hopkins Bloomberg School of Public Health, Baltimore, MD, USA.; c Breakthrough ACTION Guyana, Georgetown, Demerara-Mahaica, Guyana.; d Ministry of Health, Georgetown, Demerara-Mahaica, Guyana.

## Abstract

**Introduction:**

To manage the rapid rise of misleading information on the coronavirus disease (COVID-19) during the pandemic, the Breakthrough ACTION project developed a theory-based rumor-tracking system to inform Guyana’s COVID-19 communication campaign.

**Methods:**

The rumor-tracking project used the extended parallel processing model (EPPM) to identify and categorize rumors reflecting perceived high versus low vulnerability to COVID-19 and high versus low efficacy of engaging in recommended COVID-19 prevention behaviors. The project designed contextually relevant social and behavior change messages, called “MythBusters,” responded to rumor categories with the following objectives: (1) high perceived vulnerability and high efficacy rumors included a call to action; high perceived vulnerability and low efficacy rumors educated about effective and achievable solutions; (3) low perceived vulnerability and high efficacy rumors educated about risk; and (4) low perceived vulnerability and low efficacy rumors educated about risk and effective and achievable solutions.

**Results:**

Most rumors emanated from regions 4 and 8 (29%). Over two-thirds of the rumors (71%) recurred. Rumors were typically related to COVID-19 treatment or prevention (40%) and transmission (35%). Most rumors (48%) reflected low perceived vulnerability and low efficacy, 29% reflected high perceived vulnerability and low efficacy, 13% reflected low perceived vulnerability and high efficacy, and 10% reflected high perceived vulnerability and high efficacy. The project rapidly developed 12 MythBusters from June through December 2020 and integrated them into the national COVID-19 communication campaign, disseminated via radio, television, and Facebook. Estimates indicate that they have reached most of the target Guyanese population.

**Discussion:**

The EPPM was a particularly useful tool, giving direction to countering myths with appropriate messaging to affect relevant behaviors. The COVID-19 MythBusters provided the Guyanese public with valid and verifiable information and promoted preventive and protective behaviors.

## Abstract (precis)

We used a COVID-19 rumor classification tool to rapidly identify, synthesize, and counter misinformation during the COVID-19 pandemic and provide appropriate social and behavior change messaging that would affect relevant preventive and protective behaviors.

## Teaser

**Key Findings:**

- Misinformation and rumors propagated during the coronavirus disease (COVID-19) pandemic and ensuing infodemic confuse people and undermine the effectiveness of public health responses.
- Most of the 48 rumors that a rumor classification tool identified related to false, ineffective, or harmful COVID-19 prevention and treatment measures, inaccurate explanations of transmission, and doubts about COVID-19's existence.
- To educate individuals about COVID-19 risks and solutions, the project designed social and behavior change messages called “MythBusters,” which had an estimated reach of 30% of the population of Guyana aged 15 years and older.

**Key Implications:**

- Implementing programs can use a theoretical framework such as the extended parallel processing model during health emergencies to identify specific information content (specific inaccurate beliefs) that can be corrected if done in a timely and focused way.
- Policy makers should institutionalize social and behavior change into emergency planning to ensure proactive public health responses and resilient health systems and communities.

## INTRODUCTION

The World Health Organization (WHO) declared the coronavirus disease (COVID-19) a pandemic on March 11, 2020—the same date Guyana reported its first confirmed case of COVID-19. In response to the pandemic, the Guyanese government instituted the National COVID-19 Task Force, composed of ministries that have a role to play and led by the National Civil Defense Commission. Within the task force, the Ministry of Health (MOH) coordinated the Health Emergency Operations Center (HEOC), which is responsible for the country’s emergency measures, including contact tracing, quarantine requirements, physical distancing, and mask wearing.

Located on the northern coast of South America, Guyana has a population of 756,000 and consists of 10 administrative regions designated as numbers 1 to 10. About 40% of the Guyana population lives on the Atlantic coast within Region 4, where the capital city of Georgetown is located. About 11% live in the hinterlands (Regions 1, 7, 8, and 9),^1^ remote and hard-to-reach areas that share porous borders with Brazil and Venezuela; they typically consist of small stable communities and numerous mobile workers, such as gold miners, loggers, and migrants. Both the capital city and the hinterlands pose unique challenges in curbing the COVID-19 pandemic. The capital city and its environs are densely populated and likely to facilitate inter-regional spread, whereas the hinterland regions, with their porous borders, are likely to facilitate international spread. In addition, both settings have communication systems that provide access to a glut of confusing and misleading information from international sources.^2^^,^^3^

The emergence of COVID-19 was an extraordinary global health event that generated an immense amount of information, which emanated from many disparate sources and spread rapidly worldwide via digital and social media.^4^ That information does not always accurately describe either COVID-19 or the preventive behaviors recommended to prevent transmission.^4^ WHO has called this information phenomenon an infodemic.^5^^,^^6^ Previous research defines rumor as an act of communication containing unverified or false information and can serve as a way for a group of people to make sense of uncertainty or trauma.^7^ However, the misinformation and disinformation propagated during an infodemic, as they have during COVID-19, confuse many people, leading to distrust in public health efforts and the promotion of harmful practices that undermine the effectiveness of public health responses, particularly in lower-resource settings.^2^^,^^8^

Misinformation propagated during the COVID-19 infodemic confuses many people, leading to distrust in public health efforts and the promotion of harmful practices that undermine the effectiveness of public health responses.

To respond to the pandemic, HEOC collaborated with stakeholders and implementing partners, including the Breakthrough ACTION project in Guyana, funded by the United States Agency for International Development (USAID). The project was already working in collaboration with the Guyana MOH, the Pan American Health Organization, and the Global Fund to implement innovative evidence-and theory-based social and behavior change (SBC) interventions to support community case management of the malaria program in several regions.

The HEOC and Breakthrough ACTION Guyana project sought to develop and deliver a COVID-19 communication campaign to communities within the hinterland regions most severely affected by the outbreak. This project implemented the COVID-19 campaign over 9 months, from July 2020 to March 2021, focusing on 3 COVID-19 preventive behaviors: physical distancing, hand hygiene, and wearing masks. Regional communication coordinators in each hinterland region worked with a network of volunteers in the communities to deliver COVID-19 campaign messages, educating communities about COVID-19 and prevention behaviors.

Rapid dissemination of inaccurate information can be harmful if left unabated.^9^ For example, misinformation during the COVID-19 pandemic has resulted in fatalities^10^ and widespread vaccine hesitancy.^11^ A process for systematically identifying rumors early through community-based informants, national hotlines, or social media can allow people working in risk communication to address rumors that may hinder the public health response.^12^

The project developed a rumor-tracking system to run concurrently with the COVID-19 communication campaign to manage the infodemic emerging in Guyana. The project developed its theory-based rumor-tracking system using concepts from the extended parallel processing model (EPPM), a conceptual model frequently used to understand decision making in the face of a public health threat.^13^ The EPPM describes how rational considerations (efficacy beliefs) and emotional reactions (fear of health vulnerability) combine to determine behavioral decisions.^14^ The following constructs^15^ are central to the EPPM: fear, vulnerability (with its 2 components: perceived severity and perceived susceptibility), and efficacy (comprising self-efficacy and response efficacy beliefs). Response efficacy is the perceived effectiveness of a response in averting a threat, while self-efficacy is the perceived ability to carry out the recommended response.^15^ The EPPM also includes 2 types of responses: danger control and fear control. Fear tends to motivate action, whereas efficacy beliefs determine what actions people take when motivated.^15^ However, when fear is very strong and efficacy beliefs are very weak, some people try to avoid thinking about the threat to feel less frightened, rather than taking action to reduce the threat. Rumors and misinformation can exaggerate fears and undermine trust or confidence in viable solutions, which makes rumor tracking and response so important in the fight against outbreaks like COVID-19.^4^^,^^8^^,^^9^

This study details how Guyana’s MOH and Breakthrough ACTION Guyana used EPPM to inform a strategy to identify rumors related to COVID-19 in Guyana that could negatively affect prevention behaviors and then develop counter-messaging to overcome the effects of that misinformation.^16^ The project classified rumors based on how well they reflected or could potentially impact COVID-19 risk perception and efficacy in adopting protective behaviors or related solutions.^12^^,^^17^ Consistent with EPPM-based messaging strategies, this classification aided in the identification of message content, which indicated a need for a call to action, education, or solutions to reduce the perceived threat.^14^ The overall goal was to improve public understanding of COVID-19 and increase motivation to practice appropriate COVID-19 preventive behaviors by reducing the influence of rumors and misinformation.

The project classified rumors based on how well they reflected or could potentially impact COVID-19 risk perception and efficacy in adopting protective behaviors or related solutions.

## METHODS

**FIGURE 1:**
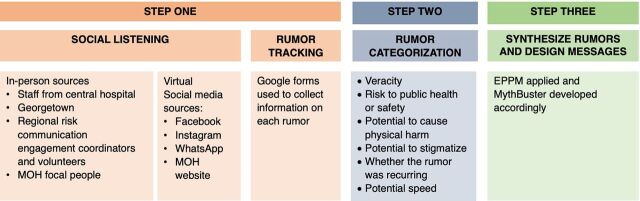
Summary of Rumor Tracking and Classification Methodology Used in Guyana Abbreviations: EPPM, extended parallel processing model; MOH, Ministry of Health.

From June 2020 to December 2020, amid projected increases in COVID-19 cases in Guyana,^18^ the National COVID-19 Task Force and Breakthrough ACTION Guyana implemented a 3-step system to (1) identify rumors, (2) categorize them using a rumor-tracking and classification tool^17^ and (3) employ the EPPM model to synthesize the rumors and develop appropriate behavior change messages known as MythBusters (Figure 1).

### Identify Rumors

The evaluators identified rumors from both in-person and virtual sources. In-person sources included regional communication coordinators, volunteer educators in each hinterland region, and medical staff at the central hospital in Region 4. These sources documented rumors heard by community members, patients, or clients. Virtual sources of rumors included social media such as Facebook and Instagram pages of national influencers and the MOH, WhatsApp messages sent to MOH staff, Breakthrough ACTION Guyana, and the MOH website.

Breakthrough ACTION developed an electronic rumor log using Google Forms based on guidance documents,^19^ and project technical briefs^20^ to track individual rumors in real time. The log documented the wording of the rumors verbatim, the location (region, district, and village), the channel of the rumor (in person or virtual), and the relevant topic (including COVID-19’s existence, source, transmission, symptoms, treatment, and prevention).

### Categorize Rumors

Second, the project assessed the logged rumors weekly for their potential risk using a second Google Form that included the following statements exploring the features of the rumors with 3-point response options:

- Veracity (true, false, or unsure) as informed by global experts, including WHO and the U.S. Centers for Disease Control and Prevention, and John Hopkins University^21^^,^^22^
- Risk to public health or safety, including the potential consequences on public opinion or response (major, moderate, or minor)
- Potential to cause physical harm or threaten lives (high, medium, or low)
- Potential to stigmatize people perceived to be at risk or infected with COVID-19 (yes, no, or maybe)
- Whether the rumor was recurring (i.e., identified and documented prior [yes, no, or do not know])
- Potential speed: rumors were classified as high speed (received/heard more than once on the same day), medium speed (received/heard more than once per week), or low speed (received/heard once per week)

Based on these responses, the project developed an 18-point risk scale. Rumors with scores of 6–8 points were determined to be low risk, 9–12 points as medium risk, and 13–18 points as high risk.

### Synthesize Rumors and Develop SBC Messages

The project used EPPM to explore perceptions related to rumors and relevant SBC messages that could address them. It categorized rumors based on whether they reflected 1 of these 2 constructs: (1) perceived vulnerability (i.e., that the rumor reflected a belief that a person or people are vulnerable to COVID-19); or (2) perceived efficacy (i.e., that the rumor reflected the belief that people feel that solutions are effective and/or that they can take action to mitigate COVID-19).

The project used EPPM to explore perceptions related to rumors and categorize the rumors based on perceived vulnerability or perceived efficacy.

**FIGURE 2:**
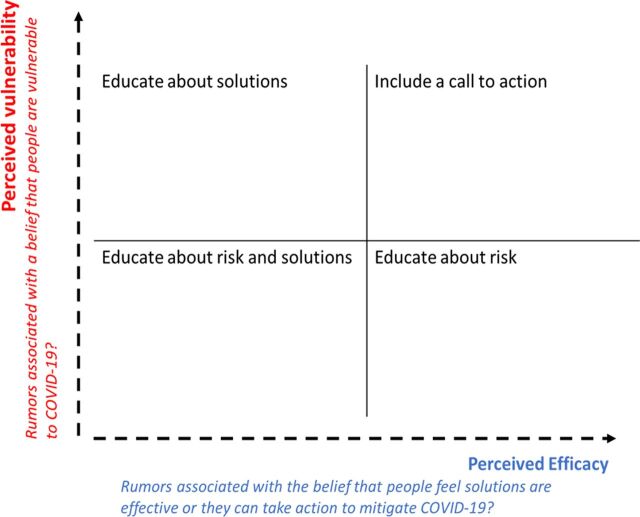
EPPM Matrix and Social and Behavior Change Message Strategies Abbreviation: COVID-19, coronavirus disease; EPPM, extended parallel processing model.

The possible response options for both questions included yes, no, and unsure. The project developed a 2-by-2 matrix of the EPPM model to classify rumors and determine the type of SBC messages to be developed (Figure 2). The program did not collect information from the population regarding their perceptions and behaviors related to COVID-19. Thus, rumors from communities were used as a proxy to understand the prevailing sentiments regarding COVID-19 in Guyana. The program explored the rumors to determine whether they reflected perceived vulnerability or efficacy in addressing COVID-19. This was done by reviewing the actual wording of the rumors, as well as any implicit meaning within the rumors. For rumors classified as having high perceived vulnerability and high efficacy, the campaign developed MythBusters, where the main thrust of the message was a call to action. Where vulnerability was present but efficacy was unlikely, the MythBuster message educated on solutions. When no perceived vulnerability existed but efficacy was present, the MythBuster message educated on the risks of COVID-19. When both perceived vulnerability and efficacy were absent, the MythBuster message educated on both risks and solutions.

The MOH and Breakthrough ACTION Guyana developed the basic message of MythBusters and selected channels for dissemination (e.g., radio, television, and social media) weekly. Custom graphics accompanied the MythBusters, and the project disseminated them using Facebook, Instagram, newspapers, and flyers. It also incorporated high-priority messages to address recurring rumors in entertainment-education “Edu-dramas” for radio or television broadcasts. MythBusters related to rumors requiring immediate action went out on the same day of development, whereas the project disseminated all other MythBusters within 1 week.

## RESULTS

### Rumors Identified

**FIGURE 3:**
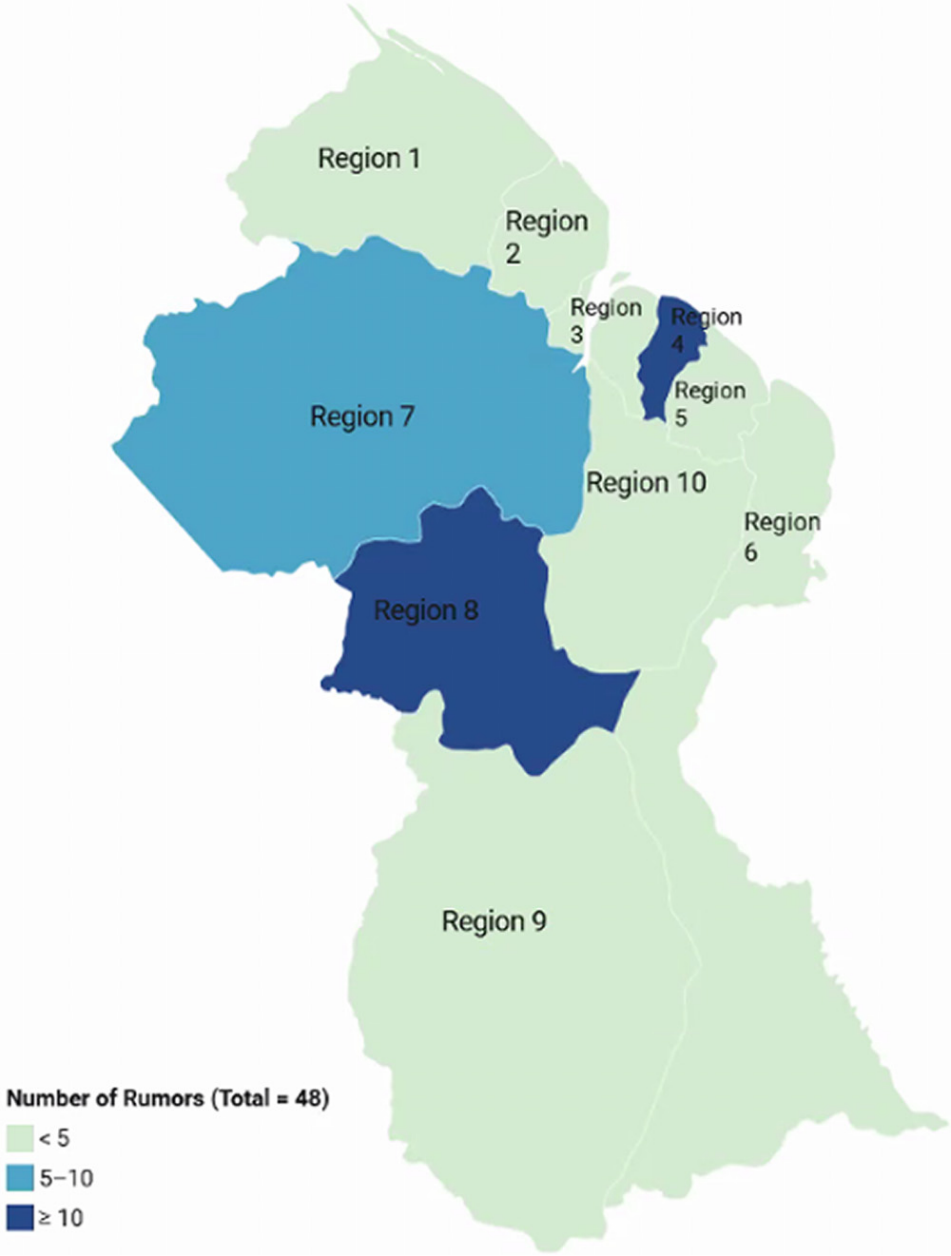
Map of COVID-19 Rumors in Guyana Abbreviation: COVID-19, coronavirus disease.

From May to December 2020, the project identified 48 rumor submissions. Figure 3 shows the number of rumors identified across the Guyana regions, and Table 1 summarizes the number of rumors by date, channel, and topic. Rumors circulated primarily from September to October (54%), with the majority emanating from Regions 4 and 8 (29% each), followed by Region 7 (17%). Most rumors came from in-person interactions (75%). Notably, risk communication and community engagement coordinators working in remote regions recorded that all rumors in these areas came from in-person sources because spread via virtual channels was unlikely or impossible. The most prevalent rumors related to false, ineffective, or even harmful COVID-19 prevention and treatment measures (40%), inaccurate explanations of transmission (35%), and doubts about the existence of COVID-19 (23%). Notably, many rumors were similar. For example, some suggested that traditional medicine, colloquially referred to as “bush” or “bush medicine,” would heal or cure and prevent COVID-19. Examples included “drink boiled papaya leaves,” “use bark and roots, and “bush medicine.” Rumors often contained more than 1 inaccuracy (e.g., “COVID-19 is a simple flu cured with ‘bush’”). At times, rumors also contained an element of truth (e.g., “coronavirus is the fancy name given to some kind of new cold” or “The only difference is that once you get the ‘Rona’ [coronavirus], and you have underlying conditions, you can die”).

**TABLE 1.** Summary of Rumors Identified in Guyana During COVID-19 Pandemic

	**No. (%) (N=48)**
Date	
May–June	1 (2)
July–August	12 (25)
September–October	26 (54)
November–December	9 (19)
Channel^a^	
In-person	36 (75)
Virtual	12 (25)
Topic^a^	
COVID-19 existence	11 (23)
Source or transmission	17 (35)
Symptoms	0 (0)
Treatment or prevention	19 (40)
Other	6 (13)

Abbreviation: COVID-19, coronavirus disease.

a Some rumors were grouped into more than 1 topic category.

Most rumors came from in-person interactions instead of social media or other virtual channels.

### Rumors Categorized

Table 2 summarizes the risk categorization of all rumors. Most rumors were classified as high risk (71%), followed by medium (21%), and low (8%). Specifically, this study found most rumors to be highly improbable or false (83%), while 52% of rumors posed a high potential to cause physical harm, and 50% of them posed a high risk to public health or safety. Almost three-quarters of the rumors recurred (71%), and a small percentage posed the risk of stigmatizing persons at risk of infection or already infected with COVID-19 (8%). Over three-quarters (77%) of the rumors were spread at a moderate (more than once a week) or high (daily) rate.

**TABLE 2.** Summary of Rumor Risk Categorization in Guyana During COVID-19 Pandemic

**Rumor Characteristic**	**No. (%)(N=48)**
Veracity	
False	40 (83)
Unsure	7 (15)
True	1 (2)
Risk to public health or safety	
Major	24 (50)
Moderate	16 (33)
Minor	8 (17)
Risk of individual physical harm or promotion of risky behavior	
High	25 (52)
Medium	16 (33)
Low	7 (15)
Risk of stigmatization	
Yes	4 (8)
Maybe	3 (6)
No	41 (85)
Recurring rumor	
Yes	34 (71)
Don’t know	5 (10)
No	9 (19)
Speed of rumor	
≥7 times per week	2 (4)
1–6 times per week	35 (73)
<1 per week	11 (23)
Overall risk score	
High (13–18)	34 (71)
Medium (9–12)	10 (21)
Low (6–8 points)	4 (8)
EPPM category	
Low vulnerability, low efficacy	23 (48)
High vulnerability, low efficacy	14 (29)
Low vulnerability, high efficacy	6 (13)
High vulnerability, high efficacy	5 (10)

Abbreviations: COVID-19, coronavirus disease; EPMM, extended parallel processing model.

### EPPM Model Applied

When analyzed according to the EPPM model, most rumors (48%) reflected low perceived vulnerability and low efficacy, 29% reflected high perceived vulnerability and low efficacy, 13% reflected low perceived vulnerability and high efficacy, and 10% reflected high perceived vulnerability and high efficacy.

### SBC Messages Designed: MythBusters

**FIGURE 4:**
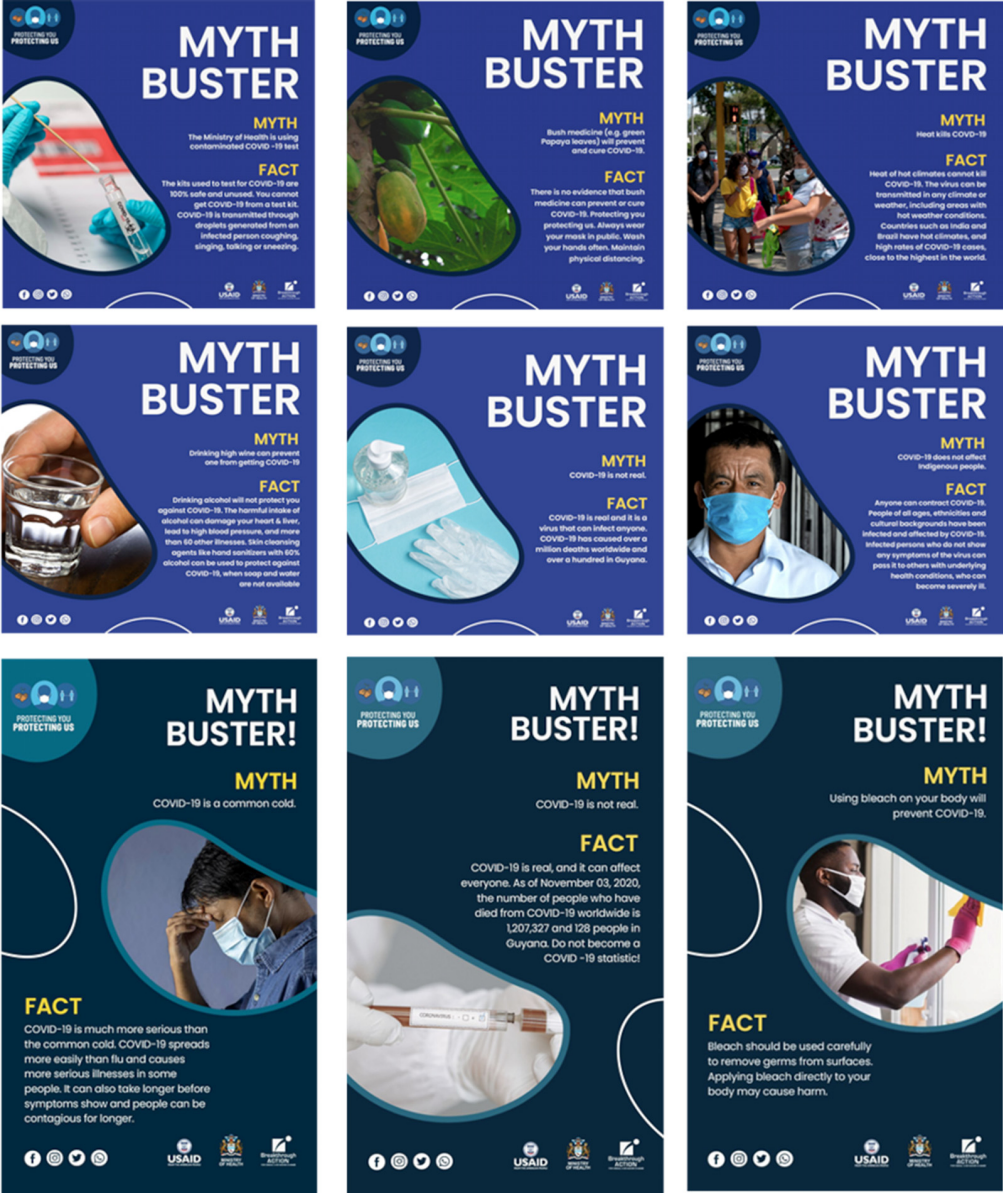
Examples of MythBusters Developed During the COVID-19 Pandemic in Guyana Abbreviation: COVID-19, coronavirus disease.

The project developed 12 MythBusters (contextually relevant SBC messages) in response to the COVID-19 rumors reported. Figure 4 highlights examples of specific MythBusters, and Table 3 highlights the EPPM classification of rumors addressed by some MythBusters. Over half (n=7) of the MythBusters required education on risk and solutions, and (n=3) required education about solutions. According to the EPPM model, 1 MythBuster educated about risk, and 1 included a call to action. The Supplement dataset includes all rumors, their characteristics, EPPM categorization, and accompanying MythBusters.

**TABLE 3.** Examples of MythBusters Developed to Address Rumors in Guyana During COVID-19 Pandemic

**MythBuster #1**	
Myth	COVID-19 does not affect indigenous people.
EPPM category	Low vulnerability, low efficacy.
Rumor examples	“COVID-19 will not affect me as I am Amerindian.”“Once you get the ‘Rona’ and you have underlying conditions, you can die.”“Only persons with underlying conditions can die from COVID-19.”
EPPM solution	Educate about risk and solutions.
Fact	Anyone can contract COVID-19. People of all ages, ethnicities, and cultural backgrounds have been infected and affected by COVID-19.Infected persons who do not show any symptoms of the virus can pass it to others with underlying health conditions, who can become severely ill.
**MythBuster #2**	
Myth	The Ministry of Health is using contaminated COVID-19 test kits.
EPPM category	High vulnerability, low efficacy.
Rumor examples	“The COVID-19 test kits are infected.”“The MOH is using testing kits that were already used to test other suspected COVID-19 cases.”“The government is giving persons COVID-19 by using contaminated nasopharyngeal swabs.”
EPPM solution	Educate about solutions.
Fact	The kits used to test for COVID-19 are 100% safe and unused. You cannot get COVID-19 from a test kit. COVID-19 is transmitted through droplets generated from an infected person coughing, singing, talking, or sneezing.
**MythBuster #3**	
Myth	Government is hiding COVID-19 numbers from Guyanese people.
EPPM category	High vulnerability, low efficacy.
Rumor examples	“Government is hiding COVID-19 numbers from Guyanese people.”“The numbers are much higher.”
EPPM solution	Educate about solutions.
Fact	The Ministry of Health publishes a COVID-19 dashboard daily for all Guyanese to see. The dashboard provides correct information about the reported COVID-19 cases and deaths in Guyana. The dashboard can be found on the Ministry of Health’s website: https://www.health.gov.gy/index.php/component/k2/item/670-guyana-covid-19-dashboard
**MythBuster #4**	
Myth	You will become infected with COVID-19 if you mix with strangers.
EPPM category	High vulnerability, high efficacy.
Rumor examples	“To prevent COVID, don't mix with strangers or persons from the coastal plains.”“Only infected persons from the coastlands can take COVID to the mining areas.”
EPPM solution	Include a call to action.
Fact	Any person with COVID-19 (even if they are not showing symptoms) can infect others, including your friends, family, or strangers. Protect yourself and your loved ones by always wearing a mask in public, washing your hands often, and practicing physical distancing (6 feet/2 meters).

Abbreviations: COVID-19, coronavirus disease; EPPM, extended parallel processing model; MOH, Ministry of Health.

The MythBusters had an estimated reach of 30% (n=151,763) of the population of Guyana aged 15 years and older. The project also integrated MythBusters into the main national COVID-19 campaign, which included educational dramas broadcast on radio, television, and Facebook, estimated to have reached the entire population aged 15 years and older. The campaign nationally disseminated the majority (95%) of MythBusters through multiple channels, including newspapers (71%), social media (57%), flyers (38%), radio (24%), and town criers (24%).

## DISCUSSION

The EPPM framework has been widely validated as a useful analytical tool in global health communication.^13^ Implementers typically use it to characterize a person’s beliefs about a health threat, what can be done about it, and the behaviors that result from those beliefs. Some beliefs can be dysfunctional or even dangerous if they lead to a person misunderstanding the true nature of a disease and making the wrong decisions—or not—about prevention and treatment.

Implementers typically use the EPMM framework to characterize a person’s beliefs about a health threat, what can be done about it, and the behaviors that result from those beliefs.

Mass media, social media, and interpersonal relationships influence individuals’ beliefs about the severity of a health threat, whether they are at risk, and how well the recommended responses work. This study used the EPPM framework to analyze the vulnerability and efficacy beliefs expressed or implied in the information people heard and saw in Guyana. Based on this analysis, we identified inaccurate information likely to spread widely, thereby influencing unhealthy thinking about COVID-19 and distrust of effective solutions. The advantage of using a theoretical framework such as EPPM to inform this analysis is that it can point to specific information content (specific inaccurate beliefs) that can be corrected if done in a timely and focused way. The framework helps to take the guesswork out of what to say in response to unscientific ideas circulating in public, redirecting the conversation to the true nature of the vulnerability and what is known to be effective in reducing that vulnerability.

Helping local implementing agencies learn to apply a framework like EPPM can improve program outcomes and sustainability. Breakthrough ACTION Guyana utilized a “learn-by-doing” approach to build the capacity of its MOH to implement the rumor-tracking system, as some members of the HEOC entered and characterized rumors and developed MythBusters in collaboration with Breakthrough ACTION. Given high Guyanese staff turnover and the need to ensure the sustainability of the rumor-tracking system beyond the life of Breakthrough ACTION’s involvement in the country, the project implemented additional training for the entire HEOC team on the development of the log system and the EPPM model.

The study findings indicate a need for sustained programmatic interventions to address existing and emerging myths and to sustain behavioral change. Transitioning the rumor system from Breakthrough ACTION Guyana to the MOH and training MOH personnel to utilize the system is a step in ensuring the sustainability of relevant interventions. Hopefully, the MOH will build on these achievements and conduct follow-up research to determine further impacts and opportunities for improvement in the program. The COVID-19 pandemic revealed the need for public health communication efforts to be more proactive. This is especially relevant given the need to address misinformation to promote COVID-19 vaccine uptake.^11^ A framework based on Ebola and Zika health emergencies—the SBC emergency helix developed by the Health Communication Capacity Collaborative Project—focuses on proactively building coordination and resilience in the health system and community.^23^ The underlying premise is that integrating SBC into emergency preparedness and response bridges health systems with the communities they serve and can change the course of an emergency. Institutionalizing SBC into emergency planning can help create conditions under which future emergencies avoid unfolding into long-term development losses because it builds resilience in health systems and communities. The framework emphasizes the “unending” cycle of emergency preparedness, response, recovery, and resilience with communities at its center.

Integrating SBC into emergency preparedness and response bridges health systems with the communities they serve and can change the course of an emergency.

This study strongly aligns with the WHO-developed public health research agenda created in response to the global infodemic to foster a coordinated and evidence-based approach to ensure universal access to reliable health information.^24^ The research agenda includes 5 focal areas: the evaluation of infodemic impacts, how to study them, what drives them, approaches to manage them better, and considerations for operationalizing new tools for both the science and practice of infodemic management. The approach described here can be considered a contextually relevant resource for rapidly identifying, synthesizing, and acting on misinformation during health emergencies. The study also provides actionable guidance on responding to and deploying SBC interventions that protect against infodemic and its harmful effects.

However, further research should aim to evaluate the effectiveness of such SBC interventions and conduct more in-depth exploration to understand what makes people and, in turn, communities resilient to infodemics. Several rumors included in this study indicated mistrust of the pandemic response and government. While a communication campaign may reduce these rumors, it does not address the underlying reasons for distrust and may be less effective. Additional research may explore the factors associated with successful infodemic management by health authorities, the media, civil society, the private sector, and other stakeholders. Such research would promote an understanding of how misinformation has affected behavior among different populations and inform the development of interventions that address individual, community, cultural, and societal factors affecting trust and resilience to misinformation. Another opportunity for future programs is to ensure bidirectional rumor-tracking systems across all channels. While this study collected rumors through multiple channels, including interpersonal and virtual sources, response efforts primarily used print and virtual mass media channels. Future programs should explore interpersonal communication strategies such as community engagement in addressing rumors as multichannel approaches, which are typically more effective. Finally, subsequent research that explores how to optimize rumor-tracking and management systems should include evaluation or impact assessments that investigate the effectiveness of the rumor management mechanism on relevant outcomes, such as improved attitudes, knowledge, and behaviors.

### Strengths and Limitations

A key strength of this study is the systematic, theory-informed approach used to identify, classify, and address rumors rapidly. In the face of a public health emergency, the project rapidly developed real-time responses to address key rumors. However, this study has some limitations. The coding of rumors using EPPM is subjective in many instances. Certain rumors did not fit neatly into the vulnerability/efficacy rubric. This study attempted to address this by having multiple people categorize and reach a consensus. However, the rationale for this categorization has not been well documented during stakeholder meetings. Additionally, some MythBusters were not always aligned with the EPPM model, particularly if a MythBuster had already been created to address the same underlying belief with a similar EPPM rating. The rationale for creating MythBusters also relied heavily on the rumor categorization form, which included the risk to public safety. Thus, the team took the initial coding into account and collaboratively developed messages that would speak to communities and resonate with them without strictly adhering to EPPM categories. The limitations of the categorization of rumors may be addressed in future studies using a standardized review process that defines the criteria for perceived vulnerability and efficacy. Reviewers can objectively review rumors and categorize them more effectively using a checklist. Furthermore, such efforts would help assess inter-rater reliability and other measures of scientific rigor.

## CONCLUSION

The EPPM-based rumor-tracking system informed the national COVID-19 communication campaign in Guyana and served as a useful tool for countering myths with appropriate messaging to affect relevant behaviors. Theory-driven SBC messages known as COVID-19 MythBusters provided the Guyanese public with valid and verifiable information and promoted preventive and protective behaviors.

## Supplementary Material

22-00071-Olapeju-Supplement.xlsx

## Notes

**Peer Reviewed**
